# Perioperative Use of Pregabalin vs. Duloxetine for Pain Management of Knee Fracture Surgery: A Double-Blind Randomized Clinical Trial

**DOI:** 10.5812/ijpr-157958

**Published:** 2025-02-11

**Authors:** Mohadeseh Masoumi, Mohammad Soleimani, Tara Shekari, Maryam Alaei, Mehrdad Sheikhvatan, Mojtaba Mojtahedzadeh, Kamal Basiri, Farhad Najmeddin, Seyyed Hossein Shafiei

**Affiliations:** 1 Department of Clinical Pharmacy, Faculty of Pharmacy, Tehran University of Medical Sciences, Tehran, Iran; 2 Orthopedic Subspeciality Research Center, Sina University Hospital, Tehran University of Medical Sciences, Tehran, Iran; 3 Department of Pharmaceutics, Faculty of Pharmacy, Tehran University of Medical Sciences, Tehran, Iran; 4 Okan University Hospital, Okan University, Istanbul, Turkey; 5 Anesthesia, Critical Care and Pain Management Research Center, Tehran University of Medical Sciences, Tehran, Iran; 6 Prehospital and Hospital Emergency Research Center, Sina Hospital, Tehran University of Medical Sciences, Tehran, Iran

**Keywords:** Analgesia, Duloxetine Hydrochloride, Knee Fractures, Pain, Pregabalin, Postoperative

## Abstract

**Background:**

Effective postoperative pain management, particularly in orthopedic procedures, presents significant challenges. There is increasing evidence supporting the benefits of multimodal analgesia, including the use of gabapentinoids and serotonin norepinephrine reuptake inhibitors (SNRIs), to minimize opioid consumption while effectively managing pain. However, a gold-standard treatment has not been established.

**Objectives:**

This study aims to compare the efficacy of duloxetine and pregabalin within a multimodal analgesic regimen for managing postoperative pain and their opioid-sparing effects following knee fracture surgery.

**Methods:**

In this double-blind randomized clinical trial (RCT), 54 patients undergoing knee fracture surgery were randomized to receive either 75 mg oral pregabalin or 30 mg duloxetine twice daily, starting at least 24 hours prior to surgery and continuing up to 48 hours postoperatively. Pain severity was assessed at admission and at 6, 12, 24, and 48 hours post-operation. Patients reporting a pain score greater than six on a Numeric Rating Scale (NRS) received intramuscular morphine. Additionally, total opioid dose, associated complications, and drug adverse effects were monitored within the first 48 hours post-surgery.

**Results:**

Although there was no statistically significant difference between the duloxetine and pregabalin groups at each time point, the reduction in pain at the 48-hour mark was more pronounced in the duloxetine group compared to the pregabalin group. The duloxetine group required higher doses of morphine on the first day compared to the pregabalin group (3.96 ± 3.20 mg vs. 2.14 ± 2.72 mg, P = 0.022). However, on the second day, opioid rescue was required in three patients in the pregabalin group, whereas no patients in the duloxetine group required rescue. No clinically significant adverse effects were observed in either group.

**Conclusions:**

Duloxetine 60 mg per day is an equally effective perioperative alternative to pregabalin 150 mg per day, resulting in a slight increase in rescue opioid administration with equivalent analgesic efficacy during the first 24 hours postoperatively. It demonstrates notable analgesic outcomes with no increased need for opioids between 24 to 48 hours post-surgery.

## 1. Background

Effective post-operative pain prevention and management, particularly in orthopedic procedures, is challenging (1). Research has demonstrated the benefits of multimodal analgesia, which targets multiple analgesic mechanisms. Evidence suggests that the adjunctive use of agents such as gabapentinoids, nonsteroidal anti-inflammatory drugs (NSAIDs), and serotonin norepinephrine reuptake inhibitors (SNRIs) can offer opioid-sparing effects. Both duloxetine (as an SNRI) and gabapentinoids have gained popularity (2). Although guidelines' recommendations are sometimes contradictory (3, 4), their use in orthopedic perioperative pain is supported by meta-analysis studies; however, the gold-standard treatment is not described (5, 6). Improper management of pain during the initial post-operative period can lead to inadvertent outcomes, including neuropsychological issues such as anxiety and potentially post-traumatic stress disorder (PTSD). Poorly treated severe acute pain within the first 48 hours after surgery is identified as a risk factor for chronic postsurgical pain (7). Experts emphasize that the perioperative period is crucial for implementing proper analgesics to accelerate patient recovery (8, 9).

Pregabalin, classified within the gabapentinoids family, has been the subject of research regarding its efficacy and safety. It works directly on the nociceptors in pain transmission by inhibiting calcium channels, with a binding affinity six times greater compared to gabapentin. Pregabalin is allowed to be administered every 12 hours, in contrast to gabapentin, which requires administration three times daily (10, 11). Despite concerns about dizziness as a common side effect during the initial period, high perioperative doses have been studied. However, there are variable results regarding its effectiveness (12, 13).

Duloxetine is another option suggested by studies to manage post-operative pain, with potentially less effect on cognition compared to gabapentinoids. Furthermore, it offers anti-anxiety and antidepressant benefits that typically manifest within a week, which could improve outcomes, including severe post-operative pain (8, 14). Duloxetine is generally considered safe, but it can have notable side effects, including insomnia and nausea/vomiting (6). Duloxetine substantially inhibits serotonin and norepinephrine reuptake and has a central and peripheral dual-modulating effect on pain (15). Unlike its psychotropic effects, duloxetine attains its peak analgesic effect within six hours following administration (16).

Although both duloxetine and pregabalin use in orthopedic perioperative pain management is supported by meta-analysis studies, there are limited head-to-head studies (5, 6, 17, 18). We hypothesized that the pain score changes from baseline in the pregabalin and duloxetine groups may demonstrate a significant difference at 6, 12, 24, and 48 hours postoperatively. In addition, the cumulative opioid usage within 48 hours postoperatively may show a significant difference between the pregabalin and duloxetine groups.

## 2. Objectives

This study aims to compare the efficacy of duloxetine and pregabalin within a multimodal analgesic plan for managing post-operative pain and their effect on reducing patients' reliance on opioids following knee fracture surgery.

## 3. Methods

This double-blind randomized clinical trial (RCT) was designed to compare the effect of pregabalin versus duloxetine as components of a multimodal analgesic strategy on acute perioperative pain and opioid consumption following knee fracture surgery. The study was approved by the Ethics Committee of Tehran University of Medical Sciences (IR.TUMS.TIPS.REC.1402.008) and registered on the Iranian Registry of Clinical Trials website (IRCT20230416057923N1). Patients admitted for knee fracture to a tertiary hospital and undergoing surgery were enrolled after informed consent was obtained. According to CONSORT reporting guidelines (19), patients were randomized into pregabalin and duloxetine groups. In addition to the intervention medications, both groups received a standard analgesic treatment, which included a combination of scheduled celecoxib, acetaminophen, and morphine sulfate as a rescue analgesic for up to the first 48 hours post-surgery.

Inclusion criteria for enrollment were knee fractures, including distal femur, proximal tibial, and patellar fractures, consent to participate, age between 18 and 80 years, and no history of chronic or recent use of duloxetine, gabapentin, or pregabalin within two weeks before enrollment. The exclusion criteria included a history of hypersensitivity to the study drugs, creatinine clearance less than 30 mL/min according to the Cockcroft-Gault equation, severe hepatic impairment as determined by liver enzyme levels greater than five times the upper limit of normal or by Child-Pugh class C, severe or moderate opioid dependence based on the diagnostic and statistical manual of mental disorders, fifth edition (DSM-5) criteria, recent opioid use within 24 hours of study enrollment, Body Mass Index (BMI) ≥ 40 kg/m², American Society of Anesthesiologists physical status IV/V, epidural or general anesthesia in surgery, pregnancy or lactation, intolerance to oral medication, active peptic ulcer, high risk of bleeding pre- or post-surgery, concomitant participation in another clinical trial, concomitant or recent use of medications with a high potential for drug interactions (St. John's Wort, monoamine oxidase inhibitors, tricyclic antidepressants, and SNRIs) within two weeks, hemodynamic instability, undergoing surgery within less than 24 hours after admission, and any communication barrier that would hinder the evaluation of patients.

Upon enrollment in the study, laboratory parameters such as complete blood count, blood sugar, creatinine, bilirubin, alkaline phosphatase, alanine transaminase, and aspartate transaminase were examined to evaluate the eligibility of participants.

An online block randomization program divided the patients into two groups. Patients were assigned to groups using a randomization list, with each patient assigned a unique code. The hospital inpatient pharmacy prepared identical capsules containing either duloxetine or pregabalin from a similar source brand and then delivered a packet of eight capsules that matched this code to the orthopedic ward, where nurses administered the capsules. This blinding process ensured that patients, prescribers, nurses, pharmacists involved in pain assessment, and those performing the final analysis remained unaware of the patients' group assignments. Only the researcher who prepared the drug envelopes knew the allocations and was not involved in pain assessment or analysis.

The patients were administered capsules containing 75 mg pregabalin or 30 mg duloxetine every 12 hours, commencing at least 24 hours before the surgery (with a minimum of two doses preoperatively). The doses were continued for 48 hours following the operation. These dose schedules were selected based on proposed perioperative dosing of duloxetine and pregabalin in previous studies, and duloxetine 30 mg every 12 hours was considered equivalent to 60 mg per day to synchronize the schedule of interventions and to protect the blinding strategy of the trial (20, 21). In addition, all patients were given pain relievers per hospital guidelines, including oral acetaminophen 500 mg every 6 hours and celecoxib 200 mg every 12 hours after the surgery. Patients were allowed a maximum of two injections of 1-gram acetaminophen during the post-operative NPO (nothing by mouth) period.

Spinal anesthesia was performed for all patients with 2.8 to 3.2 mL bupivacaine hydrochloride 0.5% heavy (Aspen) and fentanyl (Caspian) 25 µg. Vital signs (heart rate, blood pressure, respiratory rate) were monitored at the commencement of the study, before the operation, and at 6, 12, 24, and 48 hours after the operation, as well as before each drug administration. The study pharmacist evaluated the severity of pain at the time of admission and at 6, 12, 24, and 48 hours following the operation through direct interviews with the patient or over the phone, following initial in-person training. If the patient reported a pain score of more than six on a Numeric Rating Scale (NRS), intramuscular morphine at a dose of 0.05 mg/kg (up to 4 mg) was administered with a maximum frequency of every 4 hours according to the routine practice of the orthopedic physicians and orthopedic ward.

In addition to pain assessment, the total opioid dose and any associated complications and drug adverse effects, including confusion, dizziness, hypotension, hypoxia, headache, nausea, and vomiting, were closely monitored within the first 48 hours post-surgery. The sample size was calculated for each study arm based on NRS change as the primary outcome. The standard deviation of NRS change was considered 1.2 (22), and an effect size of more than 1 score was considered clinically significant (23). With a two-tailed test α = 0.05 and a power of 0.80, a sample size of 24 patients in each study arm was calculated. Allowing for a near 30% drop-out rate, 34 patients were recruited in each group.

To describe the data, frequency and percentage indices were used for qualitative variables, and mean ± standard deviation indices for quantitative variables. To check the difference between groups, Fisher's exact test or chi-square test was used for qualitative variables, the independent *t*-test was used for normal quantitative variables, and the Mann-Whitney test was used for nonparametric quantitative variables. The trend of the changes in study parameters was evaluated with a repeated measures ANOVA test. In all tests, the significance level was set at 5%, and statistical analysis was done using SPSS software, version 26.

## 4. Results

**Figure 1.**
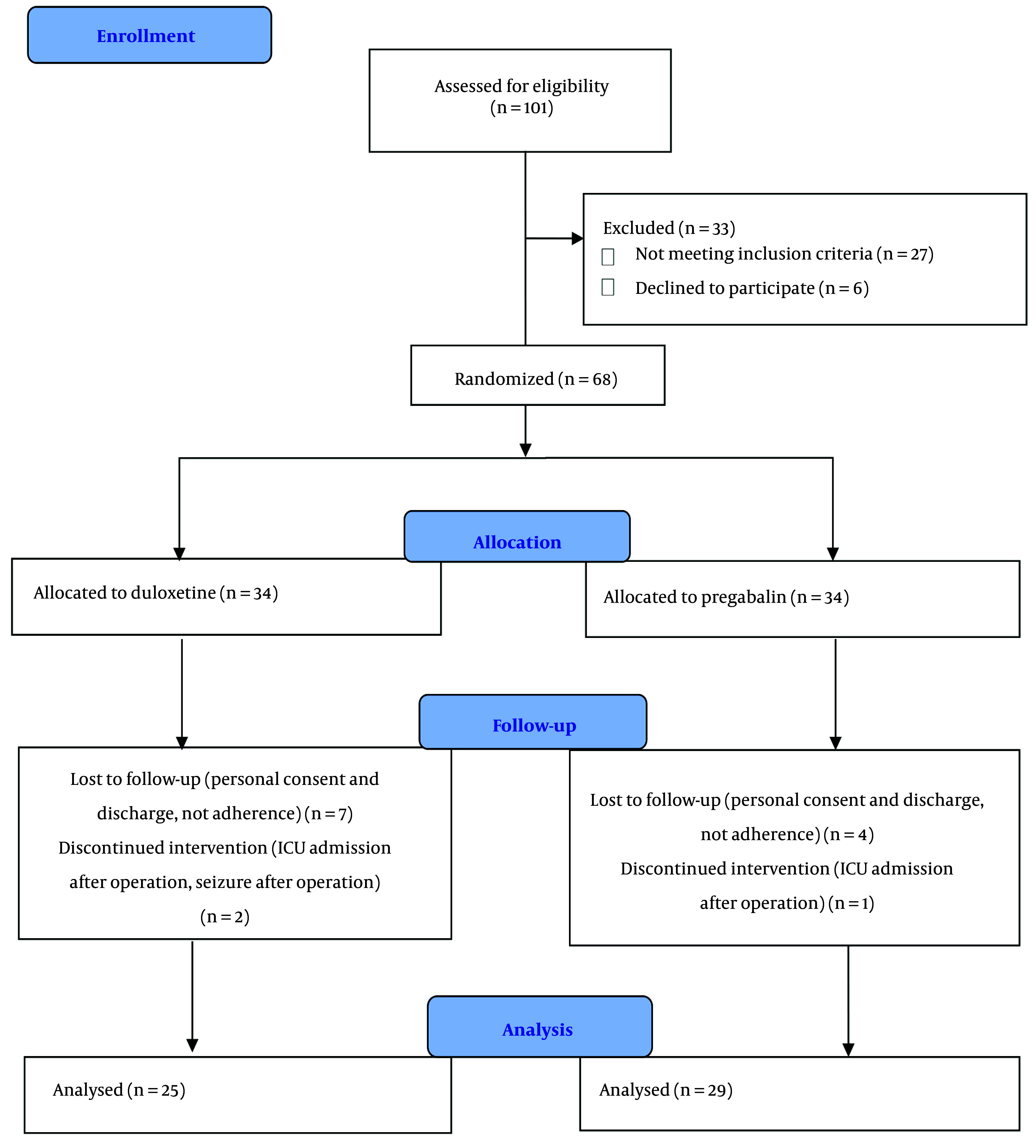
CONSORT diagram

In this clinical trial, 101 patients with knee fractures scheduled for surgery were screened for eligibility from June 2023 to May 2024. After the eligibility evaluation, 68 patients were allocated to either the duloxetine or pregabalin groups, and 54 patients completed the study (Figure 1). The demographic data and baseline patient characteristics showed no notable differences between the two groups (Table 1). 

**Table 1.** Demographic and Baseline Data ^a^

Variables	Duloxetine (N = 25)	Pregabalin (N = 29)	P-Value
**Age (y)**	45.88 ± 16.57	42.9 ± 16.02	0.505
**Gender (male)**	21 (84)	25 (86.20)	1.000
**BMI (kg/m** ^**2**^ **)**	26.45 ± 3.56	26.05 ± 3.91	0.695
**Opium addiction, mild dependence**	2 (8.00)	3 (10.30)	1.000
**Fracture location**			0.339
Distal femur	4 (16.00)	4 (13.79)	
Tibial plateau	19 (76.00)	18 (62.07)	
Patella	1 (4.00)	5 (17.24)	
Distal femur and tibial plateau	1 (4.00)	1 (3.45)	
Distal femur and patella and tibial plateau	0 (0.00)	1 (3.45)	
**Surgical approach**			0.765
ORIF	10 (34.50)	8 (32.00)	
CRIF	1 (3.40)	2 (8.00)	
**Duration of surgery (min)**	133.5 ± 46.61	126.36 ± 51.77	0.743

Abbreviations: BMI, Body Mass Index; ORIF, open reduction and internal fixation; CRIF, close reduction and internal fixation.

^a^ Values are expressed as mean ± SD or No. (%).

**Figure 2.**
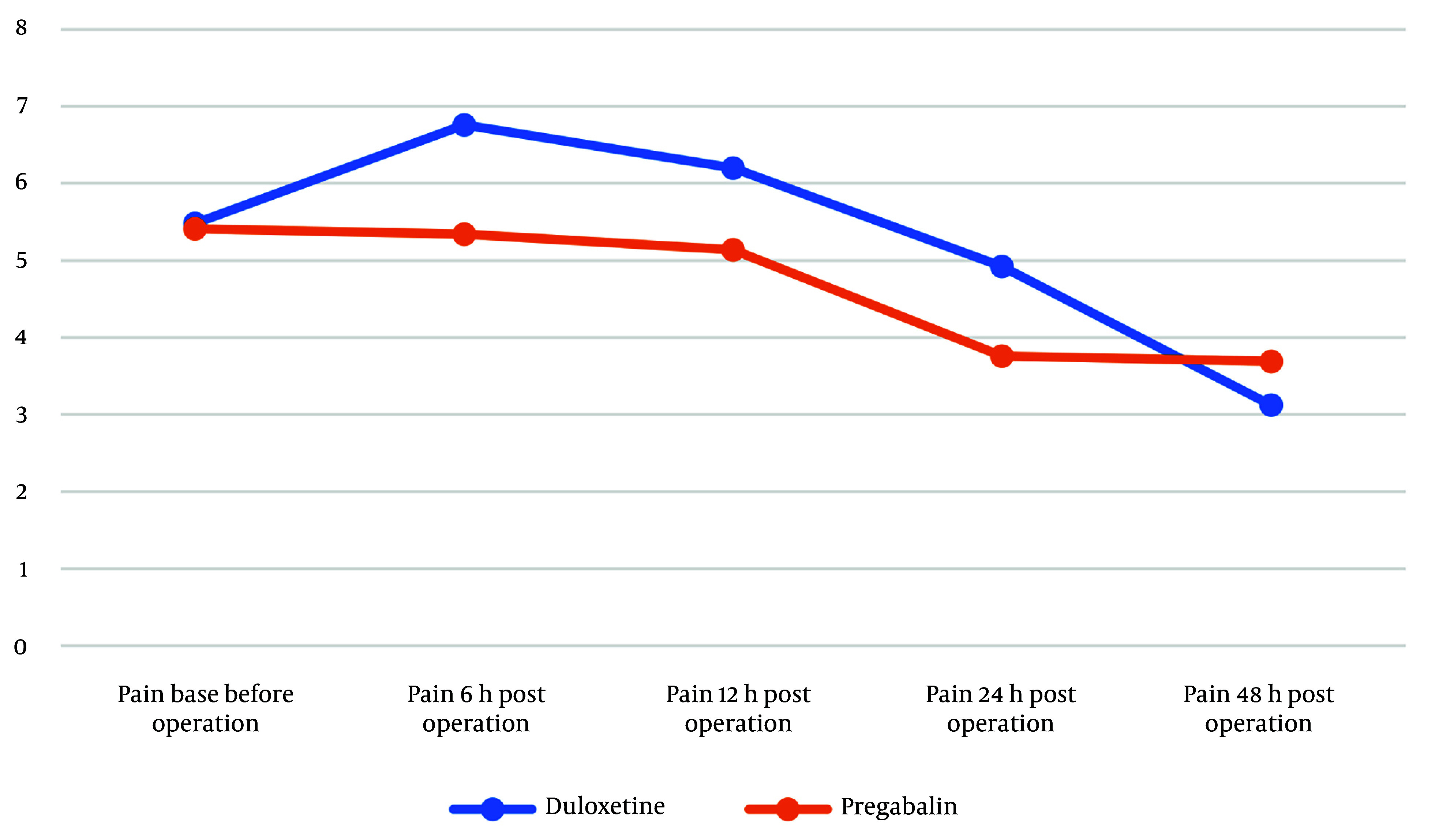
Numeric Rating Scale (NRS) score changes over 48 hours in the two groups

NRS scores at each time point and the change in pain scores at the end of the study (48 hours post-operative) compared to various time intervals are presented in Table 2. Despite the absence of a statistically significant difference between the duloxetine and pregabalin groups at each time point, the amount of pain reduction at 48 hours from any given time point was more pronounced in the duloxetine group compared to the pregabalin group. Duloxetine showed a statistically significant decrease in post-operative NRS scores compared to the pregabalin group, specifically from 24 to 48 hours (Figure 2), (NRS score changes over 48 hours in the two groups). The mean difference in pain reduction was -1.99 from 6 to 48 hours and -1.73 from 24 to 48 hours. With an effect size of more than 1 score (23), these results suggest a meaningful difference in pain reduction between the two groups. There was no meaningful difference between the two groups in each NRS score at 6, 12, 24, and 48 hours, but each period assessment showed a much more significant score reduction for the duloxetine group (Table 2). 

**Table 2.** Numeric Rating Scale Scores and Reduction in Them ^a^

Variables	Duloxetine (N = 25)	Pregabalin (N = 29)	P-Value
**NRS score at baseline **	5.48 ± 2.71	5.41 ± 3.39	0.938
**NRS score at 6 h post operation**	6.76 ± 2.96	5.34 ± 2.95	0.086
**NRS score at 12 h post operation**	6.20 ± 2.68	5.14 ± 2.75	0.158
**NRS score at 24 h post operation**	4.92 ± 3.04	3.76 ± 2.23	0.112
**NRS score at 48 h post operation**	3.12 ± 2.40	3.69 ± 2.71	0.421
**Difference between NRS score from baseline to 48 h**	-1.04 ± 3.28	-0.07 ± 3.13	0.565
**Difference between NRS score from 6 to 48 h**	-3.64 ± 3.30	-1.65 ± 2.63	0.011
**Difference between NRS score from 12 to 48 h**	-3.08 ± 2.58	-1.45 ± 2.92	0.023
**Difference between NRS score from 24 to 48 h**	-1.80 ± 2.47	-0.07 ± 1.87	0.007

Abbreviation: NRS, Numeric Rating Scale.

^a^ Values are expressed as mean ± SD.

When rescue opioid use was compared between the two groups on the first and second post-operative days, the duloxetine group received higher doses of morphine on the first day compared to the pregabalin group (3.96 ± 3.20 mg vs. 2.14 ± 2.72 mg, P = 0.022). However, opioid rescue on the second day was required in three patients in the pregabalin group, whereas no patients in the duloxetine group required it. Regarding adverse effects, two patients in each group reported nausea and vomiting, and one patient in the pregabalin group developed hypotension 48 hours after surgery. No significant adverse effects were observed, and hemodynamic parameters were similar and within normal physiological values at all times in both groups.

## 5. Discussion

Duloxetine use was associated with more intensive pain reduction at 48 hours compared to pregabalin. Nevertheless, the duloxetine group's higher pain scores in the initial phases of the post-operative period, despite their lack of significance, and their increased need for opioid rescue dosages on the first post-operative day, indicate a delayed onset of analgesic effects compared to pregabalin. These results suggest more effective acute pain management with duloxetine than pregabalin in knee fracture surgery, although it may be associated with a 24-hour delay in peak effects. Post-operative pain is classified into acute pain, lasting up to 7 days post-surgery, and chronic pain, which persists beyond 3 months. Effective acute pain management reduces morbidity and enhances recovery by shifting from opioid-based therapies to multimodal analgesia; however, more research is needed on the efficacy and safety of newer adjuvant analgesics (24).

There is limited evidence regarding duloxetine and pregabalin use in the setting of acute post-operative pain management. Several systematic reviews and meta-analyses have reviewed the effect of duloxetine on post-operative pain; however, high heterogeneity in the studies regarding surgery type, patient population, and studied doses prevents high grading of recommendations assessment, development, and evaluation (GRADE) evidence (6, 21, 25). Subgroup analysis in the latest meta-analysis, which included 14 studies focused on orthopedics, recommended duloxetine based on low to moderate evidence over placebo (26). Several systematic reviews and meta-analyses regarding gabapentinoids, including pregabalin, reached similar conclusions and limitations as duloxetine, with low to moderate evidence to recommend pregabalin over placebo (13, 27, 28).

There are few head-to-head comparisons of duloxetine and pregabalin, including only three randomized controlled trials conducted in different surgical settings. In 2018, Altiparmak et al. compared the effects of pregabalin and duloxetine on pain management and cognitive function following lumbar disc herniation surgery in 99 patients divided into three groups: Pregabalin, duloxetine, and placebo. Mean point-to-point pain scores within 48 hours after surgery were not significantly different between pregabalin and duloxetine, although both were more effective than placebo, and the pregabalin group experienced more severe cognitive impairment. The researchers concluded that duloxetine might be an excellent analgesic alternative to pregabalin for providing post-operative pain relief after spinal surgery, with similar analgesic effects but less adverse impact on the cognitive function of the patient (29).

Chachra et al. conducted a randomized controlled trial (RCT) on 60 patients undergoing surgery for lower limb trauma, who were prescribed either duloxetine or pregabalin. The rate of rescue analgesic consumption was not significantly different between the two groups in the first 72 hours after surgery. Pain scores and total rescue analgesics consumed were similar for both groups. The researchers in this study suggested that, despite numerous studies showing that a single dose of pregabalin or duloxetine can reduce post-operative analgesic requirements, prolonged administration may be necessary for optimal analgesic efficacy (30).

Imani et al. conducted a three-arm RCT on 60 patients undergoing total knee arthroplasty surgery who received either pregabalin, duloxetine, or placebo. Both pregabalin and duloxetine showed equal efficacy in pain relief and reduction in the consumption of analgesics in the early post-operative period following knee joint replacement surgeries (22). In these studies, despite differences in the baseline analgesic regimen, rescue analgesics, and variations in the type of surgery, the effectiveness of duloxetine and pregabalin was similar at 48 hours post-surgery. Conversely, in the current trial, more effective pain reduction at 48 hours in the duloxetine group suggests enhanced analgesic benefits with no increased need for rescue opioid use throughout the second day post-operation. These observations regarding the difference in the delayed phase after surgery at 48 hours may be attributed to differences between the two drugs in the time taken to reach peak effects. Such findings have been described in the literature from other studies that observed a delay in the peak effects of duloxetine versus placebo (31).

There are several limitations in this study, including: The short post-operative follow-up, which prevents evaluation of the difference over a more prolonged period; the assessment of multiple factors (e.g., age, sex, mechanism of trauma) on the pain reduction difference among the groups was not performed due to the small sample size; and the small sample size and short follow-up period limit the generalizability and depth of the findings. We suggest further studies to evaluate more prolonged periods of acute post-operative pain and the effects on more chronic outcomes.

In summary, duloxetine 60 mg serves as an equally effective perioperative alternative to pregabalin 150 mg, resulting in a small increase in rescue opioid administration with equivalent analgesic efficacy during the first 24 hours postoperatively, while demonstrating notable analgesic outcomes with no increased need for opioids within 24 to 48 hours. This study's findings support the effectiveness of duloxetine in knee fracture surgery perioperative pain management.

## Data Availability

The dataset presented in the study is available on request from the corresponding author during submission or after publication. The data are not publicly available due to institute policy.

## Clinical Trial Registration Code:

IRCT20230416057923N1.
